# A latent highly activity energetic fuel: thermal stability and interfacial reaction kinetics of selected fluoropolymer encapsulated sub-micron sized Al particles

**DOI:** 10.1038/s41598-020-80865-2

**Published:** 2021-01-12

**Authors:** Huixin Wang, Hui Ren, Tao Yan, Yaru Li, Wanjun Zhao

**Affiliations:** State Key Laboratory of Explosion Science and Technology, Beijing Institute of Technology, Beijing, 100081 China

**Keywords:** Metamaterials, Mass spectrometry

## Abstract

Aluminum can enhance heat release of energetic composite in theory. However, the commonly used micron aluminum powder has several short comings like incomplete reaction and low reaction rate. Meanwhile, outer oxide shell of nano Al particle is thicker than micro Al, which leads to low active aluminum content and insufficient heat release. On the basis of previous research, reported fluoropolymers modified Al particles were compared and suitable F2311was chosen. Sub-micron scale Al (median particle size around 200 nm) was regarded as optimum coated object in consideration of activity content of aluminum powder changing with particle size. The super fine Al powder was prepared by electrical explosion method, and encapsulated in situ by selected fluorine rubber F2311. The experiments on thermal stability demonstrated F2311 coating thickness should be no less than 3.6 nm. These results were further confirmed by EXPLO5 thermo dynamic calculation. Calculated results showed that reaction characters of F2311 encapsulated Al exceeded conventional nano Al regardless of combustion and explosion. Scanning electron microscopy (SEM), transmission electron microscopy (TEM), laser particle size analyzer and X-ray photoelectron spectroscopy (XPS) were used to characterize coated products’ morphology, particle size distribution and interfacial bonding information. The results showed that the coated samples were generally spherical shape, with median particle size of 217.7 nm and coating thickness of 3.6 nm. The coating shell contained a small amount of alumina and aluminum fluoride besides fluoropolymer. The non-isothermal dynamic equations of Al/F2311 and Al/Al_2_O_3_ were deduced by TG/DSC simultaneous thermal analysis. Compared with conventional nano-Al, the apparent activation energy of Al/F2311 decreased by 45 kJ/mol and the first exothermic peak temperature was about 10 °C earlier. Moreover, heat release was nearly twice as conventional nano-Al. TG-DSC-MS coupled measurements certified that active Al was enveloped by ‘fluorine atmosphere’ while F2311 decomposed in range of 200–400 °C. Alumina was replaced with aluminum fluoride inside coating layer during 400–550 °C, which broadened the diffusion path and then accelerated the permeation of oxidizing gas. In addition, the exothermic of Al-F was obviously larger than Al-O. Consequently, the oxidation reaction was activated rapidly, especially in initial exothermic period. Fluoropolymer encapsulated sub-micron sized Al was a latent highly activity energetic fuel and a potential candidate for aluminum powder.

## Introduction

Aluminum has well known advantages of high oxidation heat release, low cost and abundant sources, which is often used as fuel in energetic compositions. Massive engineering practices showed that micron-sized aluminum powders are often used as metal fuel to adjust the oxygen balance of composite energetic materials which will have ideal thermodynamic properties. However, actual reaction kinetics of aluminized energetic materials is not good effect, resulting in insufficient energy release and waste of energy storage volume. Dense lattice structure of alumina on metal core’s surface hinders the diffusion of oxidizing gas. It was well known that the larger size of micron aluminum particles, the longer path of heat conduction. Undoubtedly, aluminum powder will form alumina during oxidation process which has very high phase transition temperature (melting point 660 °C, boiling point 2980 °C). Slow heat transfer rate and inert oxide shell are two main reasons why aluminum powder cannot be sufficient reaction.

If we replace micron aluminum powder with nano aluminum powder, reaction rate should be accelerated theoretically because nano aluminum has the characteristics of obvious kinetic advantage due to its small size and high specific surface area. However, nano Al particles are easily oxidized, and then oxide shells are much thicker than that of micron Al particles. Furthermore, active aluminum content decreases greatly with the reduction of particle size^1^. For example, 50 nm aluminum particle has nearly 5 nm thick oxide layer outside, and its active aluminum content is only about 42%^2^. Therefore, using nano Al powder directly cannot solve the problem of low energy release in engineering applications. Chakraborty and Zachariah^3^ believed that nano aluminum particle would agglomerate and form micron particles during combustion. The reduction of particles scale will cause the composite’s viscosity rising instead of improving reactivity. Huang et al.^4^ and Sundaram et al.^5^ considered that burning rate of aluminum powder was different with particle sizes. While particle size of aluminum powders reduces from micron to nano scale, burning rate will increase. Sundaram et al.^6^ proposed that reaction of aluminum powder is controlled by gas diffusion while particle size is larger than a certain critical diameter. On the contrary, reaction is controlled by chemical kinetics. Is there an optimal size region for Al particle in energetic composite? Assumed optimal size being, what was the matching relation between particle size and energetic composite? How can we fabricate a new fuel with excellent reaction kinetics and high combustion heat? These are the focuses in the design of energetic system^7^.

In order to improve active metal content, researchers tried to modify aluminum powder such as passivation coating and in situ coating^8^. The passivation coating was safe to operate because coating agents were covered on oxide layer^9^. Therefore, this process could not solve the problem of active aluminum content’s decline. In situ coating method was directly encapsulated nano Al particle to prevent from air oxidation, resulting in active aluminum content promotion. Up to now, in situ coating method was mostly used to modify the surface of nano Al particles.

Many coating agents have been reported, including carbon^10^, aluminum boride^11^, transition metals^12^, organic acids^13,14^, polymers^15,16^, and binder^17,18^ etc., which almost have a negative effect on energy release. It was noticed that fluoride reacts with Al to generate AlF_3_, and its exothermic value is 55.66 kJ/g, which is much higher than formation heat of Al_2_O_3_ (30.95 kJ/g)^19^. Meanwhile, fluoropolymer with good compatibility and stability was promising to protect active metal and improve energy output, which was commonly used as a binder to mix energetic composite. At present, polytetrafluoroethylene (PTFE)^20^, polyvinylidene fluoride (PVDF)^21^, perfluoropolyether (PFPE)^22–24^, perfluorotetradecanoic acid (PFTD)^25^ and perfluorohexadecanoic acid (PFHD)^26^ have been studied to coat nano Al particles. However, there were controversies and even opposite conclusions about the influence of fluoropolymers on the energy release of nano Al. Published data were derived from different fluoropolymers, ignition modes and experimental conditions in various articles. For example, Yang et al.^27^ ignited nano Al coated by PVDF with bridge wires in unconstrained states, and then they found that ignition delay time was shortened with mass fraction of PVDF increasing^27^. Watson et al.^28^ ignited PTFE/Al composite with metal wire without constraints, while content of PTFE was less than 20%, composite could not even generate self-sustaining combustion. Under confined conditions, its burning rate could be risen by nearly 200 times^28^. They believed that gas generated by PTFE decomposition would escape and could not effective react with Al in open system. Sealing reactants can speed up reaction rate and fluorine react with the alumina shell fully, thus oxidation of Al was quickly activated resulting in a higher combustion rate. Yao et al.^29^conducted laser ignition experiments on PFTD modified nano Al. The results showed that ignition delay period of modified nano-aluminum powder shortened under low heat flux. Authors believe that thermal diffusion rate was greater than heat accumulation rate, while PFTD with low thermal conductivity was inclined to heat accumulation increasing. Under high heat flux, thermal diffusion rate is less than heat accumulation rate, slow heat accumulation of PFTD led to ignition delay. Osborne and Pantoya^31^ studied the reaction of alumina shell and PTFE. They found that an exothermic pre-ignition reaction (PIR) between PTFE and Al_2_O_3_ occurred before the oxidation of aluminum core. It reminded us that PTFE maybe removed oxide layer to release heat, and then stimulate the oxidation process of Al core. They also noted that PIR phenomenon is not obvious on micron aluminum powder. Some researchers also used TG/DSC/MS to study the thermal decomposition behavior of Al/PVDF in inert gas^32,33^. The results showed that PIR appeared about 400 °C, and decomposition of PVDF would produce HF, C, CF, CH_2_F, C_2_H_2_F, CHF_2_, C_2_H_2_F_2_ and other small molecules.

From the prior literature, it can be seen that nano aluminum modification has always been a research hotpot. PTFE and PVDF were widely used fluoropolymer to modify nano Al. Inert gas was often used to study the thermal response behavior involved by reported, which was inconsistent with the actual use state. Moreover, combustion mechanism of modified products was a disputed topic resulting from lack of experimental standards and measurement methods. In order to study the interfacial stability and thermal reaction process of fluoropolymer @ Al composite, based on previous researches, almost all of fluoride materials coated nano Al were compared and the most suitable one was selected in this article. The optimum size range of Al powder to be coated was determined by thermodynamic calculation. Nano Al was prepared and encapsulated by fluoropolymer with one pot method. The critical thickness of the coating was obtained by thermal stability experiment.

We used TG/DSC/MS coupled equipment to investigate the reaction details on the interface of fluoropolymer @ Al composite. Kinetic equations were derived and thermal response differences between fluoropolymer @ Al and ordinary Al particles were compared. Finally, interface reaction of fluoropolymer @ Al was discussed.

## Experimental

### Selection of fluoropolymer coating

Fluoropolymer with high fluorine content include polytetrafluoroethylene (PTFE), polyvinylidene fluoride (PVDF), F23 series and F26 series. F23 series fluororubber is a kind of copolymer of vinylidenefluoride–trifluorochloro ethylene, such as F2311 (vinylidene fluoride and trifluorochlor oethylene ratio is 1:1), F2314, F2319 etc. F26 series rubber is a class of vinylidenefluoride- hexafluoropropylene copolymer, including F2602, F2603 etc. (different grades represent different molecular weights). In addition, perfluoropolyether (PFPE), perfluorotetradecanoic acid (PFTD), and perfluorohexadecanoic acid (PFHD) were also involved in this literature. As well known to us, proportion of aluminum powder can be added up to 30% in energetic material formulation. As its coating agent, fluoropolymer should have certain flexibility, be beneficial to the reduction of mechanical sensitivity, have tensile strength to ensure the mechanical properties of the formula, have a moderate thermal decomposition temperature, not only to ensure the safety of storage, but also to avoid high temperature blocking the oxidation of aluminum. It should be specially pointed out that the coating experiment is carried out in the liquid phase environment, so it is necessary to investigate the solubility of coating agent. Some polymers with high fluorine content but poor solubility, such as PTFE, should be excluded. Table1 lists all the fluoropolymers mentioned in published literature to modify nano Al particles. The data in the table can clearly compare the strength, flexibility, oxidation element content, solubility and thermal decomposition temperature of various fluoropolymers. Considering the above factors, F2311 is suitable to be used as coating material for Al particles.

**Table 1:** Comparison of physical and chemical properties of various fluoropolymers.

	PTFE	PVDF	F2311	F2314	F2602	PFPE	PFTD	PFHD
Tensile strength/MPa	27.6	36–60	20.4	29.4	9.81–15.7	–	–	–
Elongation at break/%	238	50–100	410	100–300	210–230	–	–	–
Meltingpoint/°C	~ 327 (melting)	~ 170 (melting)	Direct decomposition	Direct decomposition	Direct decomposition	Liquid at room temperature	130–135 (melting),275.7 (boiling)	154–155 (melting), 304.5 (boiling)
Thermal decomposition temperature/°C	508–538	350	200–400	200–400	≥ 400	270–300	–	–
Molecular formula	(CF_2_–CF_2_)n	(CH_2_–CF2)n	(CF_2_–CFCl)m–(CF_2_–CH_2_)n (m: n = 1:1)	(CF_2_–CFCl)m–(CF_2_–CH_2_)n (m: n = 4:1)	(CF_2_–CH_2_)m–(CFCF_3_–CF_2_)n (m: n = 1:2)	(CF_2_–O–CF_2_)n	C_14_HF_27_O_2_	C_16_HF_31_O_2_
Fluorine content/%	76.0	59.4	52.6	50.2	73.1	65.5	71.8	72.3
Content of Oxidant groups (O,F,Cl)/%	76.0	59.4	72.3	77.0	73.1	79.3	76.3	76.3
Solubility	Almost insoluble in all organic solvents	Soluble in *N*-methyl pyrrolidone, dimethyl acetamide, *N*,*N*-dimethyl formamide	Soluble in low–molecular ketones and esters	Soluble in a mixture of acetone, toluene and butyl acetate	Soluble in low–molecular ketones and esters	Liquid at room temperature	Soluble in ether	Soluble in ether
Mixing with Al powder	Chemical vapor deposition, mechanical grinding	Injection molding, electrospray deposition, shock-gel process	No report	No report	Electrical exploding wires	Planetary mixer, electrostatic spinning	Mechanical mixing	Mechanical mixing
Combustion characteristic	burning rate of Al/PTFE2.5 cm/s(unconfined)752 m/s(confined)	Al/PVDF: burning rate: 1269 m/s (confined)	No reports so far	No reports so far	Al/F2602: combustion heat of F2602 coated nano-Al powder was higher than that of nano Al	Al/PFPE burning rate: 45 cm/s (confined) 2.17–5.80 mm/s (unconfined)	Al/PFTD/MoO3 burning speed: 500 m/s (confined)	Al/PFHD: burning rate:1.5 times Al (unconfined)
Experimental environment of combustion	Air	Air	–	–	Oxygen bomb	Air	Air	Air
Reference	2034	212,735	36	36	37	222,324	25	26

### Al particle size to be coated

**Figure 1:**
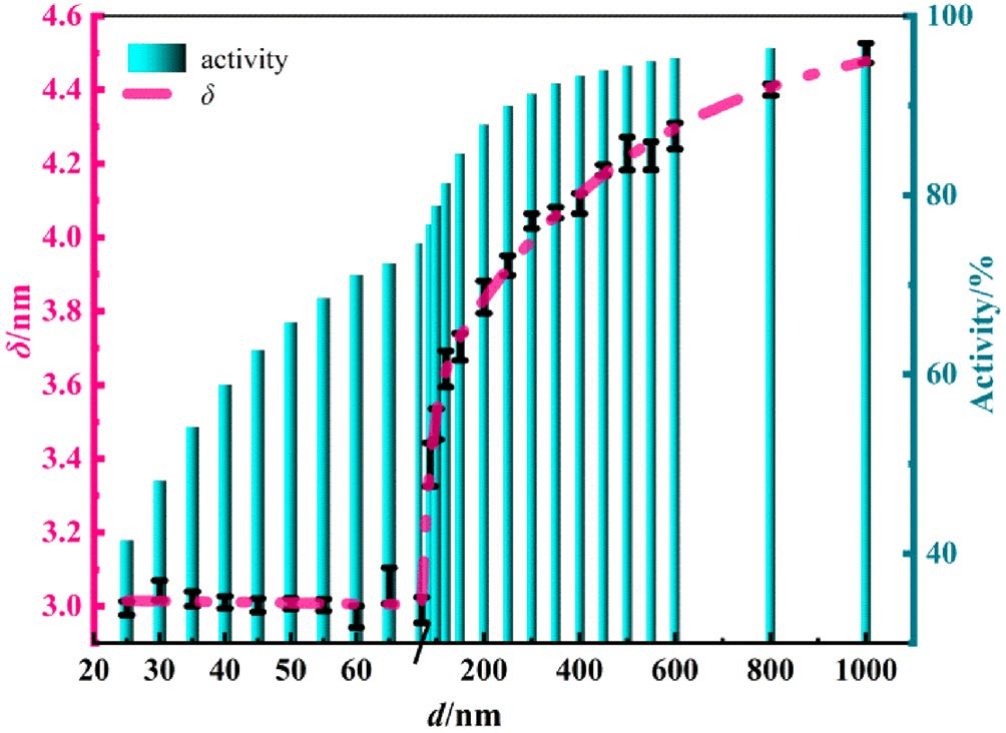
Relation between the thickness of oxide shell and particle size of Al particles.

There are different thickness of oxidized shell on the aluminum surface. And the phase transition temperature of oxidized shell is higher than that of aluminum. The structure analysis of aluminum powder shows that the oxide layer is mainly composed of octahedral Al(O_1/6_)_6_ and tetrahedral Al(O_1/4_)_4_. The average density of the oxide layer is 2.98 g/cm^3^, about 3/4 of crystal Al_2_O_3_^38^. The active aluminum content can be calculated combined with the pure aluminum density (2.70 g/cm^3^). After processing by Origin, the relationship among diameter *d*, the oxide thickness *δ* and the active aluminum content can be described according to the results of TEM, as shown in Fig. 1. Piecewise function relations between aluminum oxide layer thickness and particle size can be seen from Fig. 1. After fitting (Appendix A), the correlation of shell thickness and particle size of alumina can be obtained.

In Fig. 1, the thickness of the oxide layer is about 3 nm when *d* ≤ 70 nm. The oxide layer’s thickness is approximately exponential with the particle size when *d* is between 70 nm and 1 μm. The thickness and active aluminum content both increases rapidly at first and then slows down after *d* exceeding 200 nm. Thus, it is speculated that submicron aluminum powder of 200 nm has both high active aluminum content and high reactivity like nano powder. Therefore, in this paper, aluminum powder with a median particle size of 200 nm was selected for encapsulation in situ.

### Instruments and materials

Instrument: microphotographs were taken using a scanning electron microscope (SEM, SU8020, Hitachi, Japan) to observe the morphology of the samples. High resolution transmission electron microscopy (HTEM, Tecnai G2 F20, FEI, USA) was used to characterize the core–shell structure. To measure the particle size of samples, the dynamic light scattering nanometer particle size analyzer (Nanotrac Flex, Microtrac, USA) were used. The X-ray photoelectron spectrometer (XPS, Thermoescalab 250Xi) was used to analyze the chemical state of elements on the sample’s surface. The thermal reaction kinetics and the thermal fragments during the reaction were characterized by the simultaneous thermal analyzer-quadrupole mass spectrometry linkage device (TG-DSC-MS, STA449F3TG-DSC, QMS403D, NETZSCH, Germany).

Materials: ethyl acetate, ethyl alcohol (Sinopharm Chemical Reagent Beijing Co., Ltd), F2311 (Zhonghao Chenguang Research Institute of Chemical Industry Co., Ltd., Sichuan, China), F2311 encapsulated sub-micron sized Al (SiChuan Hbst. Co., Ltd., Sichuan China).

### In-situ encapsulation

**Figure 2:**
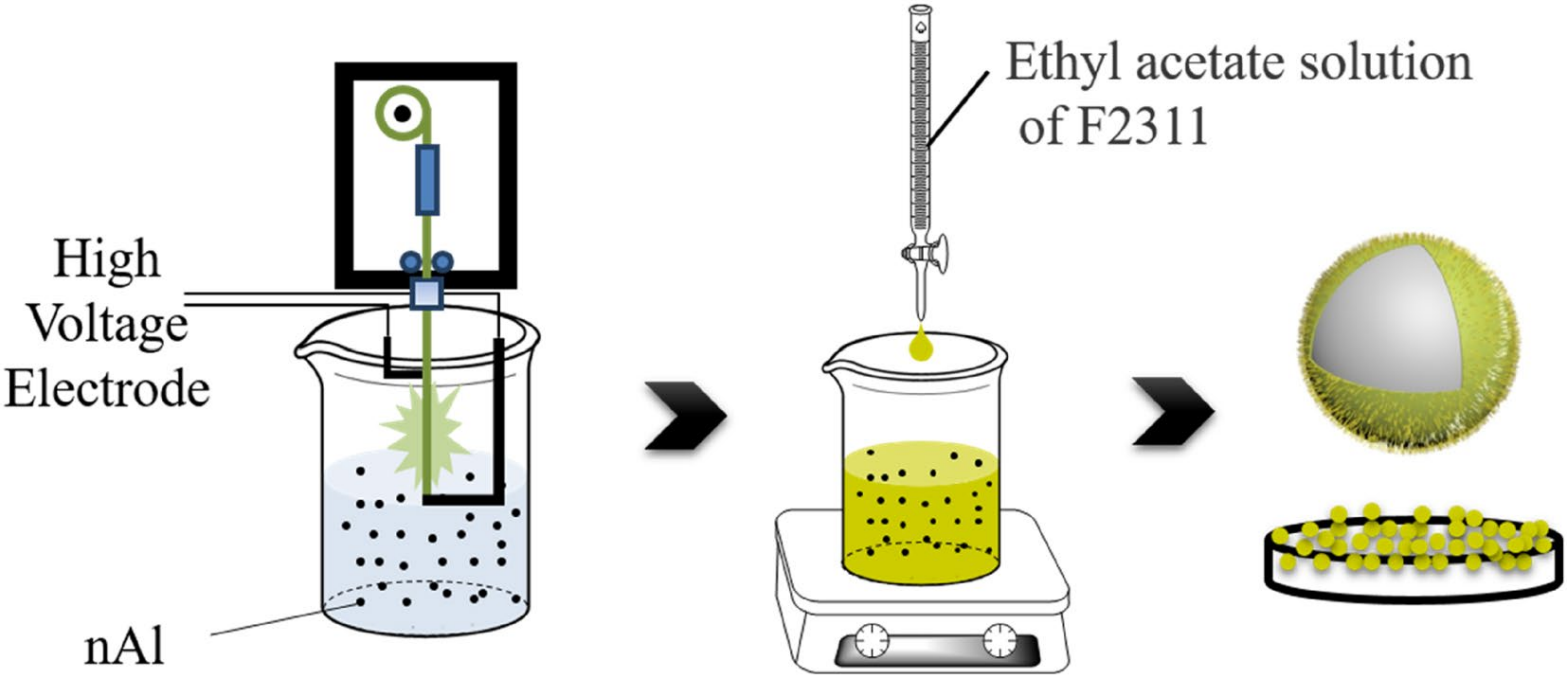
Preparation process of F2311 encapsulated aluminum powder.

The preparation process of F2311 encapsulated submicron aluminum powder is shown in Fig. 2: firstly, the submicron aluminum powder was produced using electrical exploding wires method. The pulse power generator provided an instant high voltage (1.5 kV), and then the aluminum wire (diameter 0.2 mm) in the explosion chamber vaporized due to the electric explosion. The explosion chamber was filled with argon gas, in which aluminum vapor cooled down and gathered to form submicron particles. The product was collected by separating—buffer tank—powder collection device, then directly dispersed into organic solvent. A certain amount ethyl acetate solution of F2311 was added while stirring. Changing the weight ratio of F2311 and aluminum powder would gain products with different thickness of shell. The solvent was dried at 60 °C to gain the F2311 encapsulated submicron aluminum powder. The final product is black powder. Ordinary aluminum powder was prepared by electric explosion as control under the same conditions.

## Results and discussion

### Morphology and particle size

**Figure 3:**
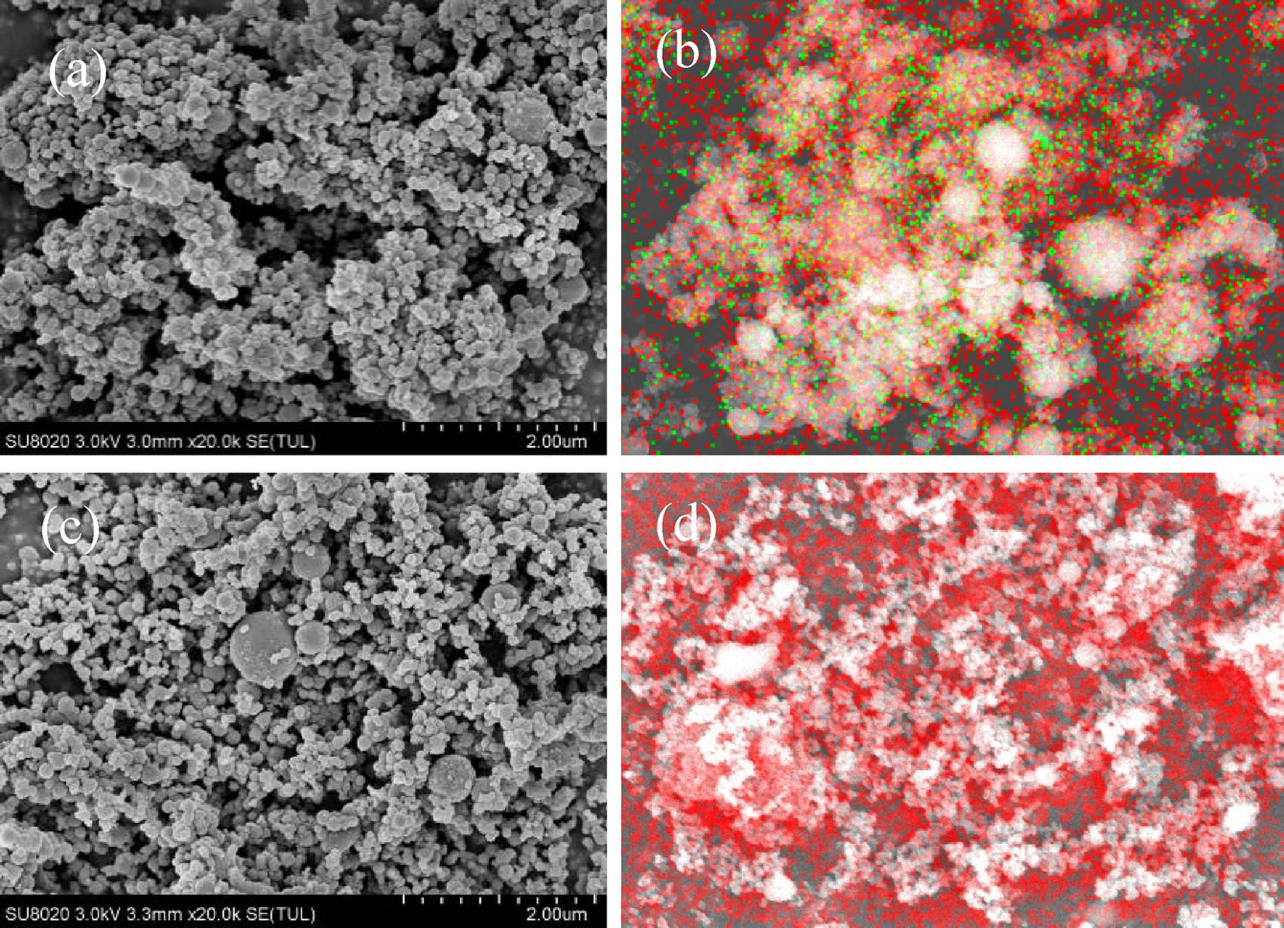
SEM and EDS photo of aluminum powder: (**a**) SEM photo of F2311 in situ coating aluminum powder; (**b**) EDS image of F2311 in situ coating; (**c**) SEM photo of raw aluminum powder; (**d**) EDS image of raw aluminum powder (green is F, red is O).

SEM images were captured and coupled with EDS mapping results to characterize the micro-structural differences between F2311@Al and ordinary Al. As shown in Fig. 3a, the F2311 @Al is spherical with different sizes ranging from 1 to 400 nm. 70% of particles are within 200 nm, but there were also 2% of particles are above 400 nm, which was caused by the unstable voltage during the electrical exploding. In terms of morphology, there is agglomeration and adhesion on the encapsulated samples compared with raw aluminum powders, which is due to the presence of polymer on the surface. Figure 3c is the SEM image of raw aluminum powder we can see that the F2311 coated aluminum powder remains spherical after the encapsulation just like raw aluminum powder. Fluorine (F) distributes evenly on the F2311@Al’s surface according to Fig. 3b. Oxygen (O) existed on the surface of both samples, indicating that there is oxide on both samples. However, the O content of the encapsulated samples is lower comparing Fig. 3b with d.

**Figure 4:**
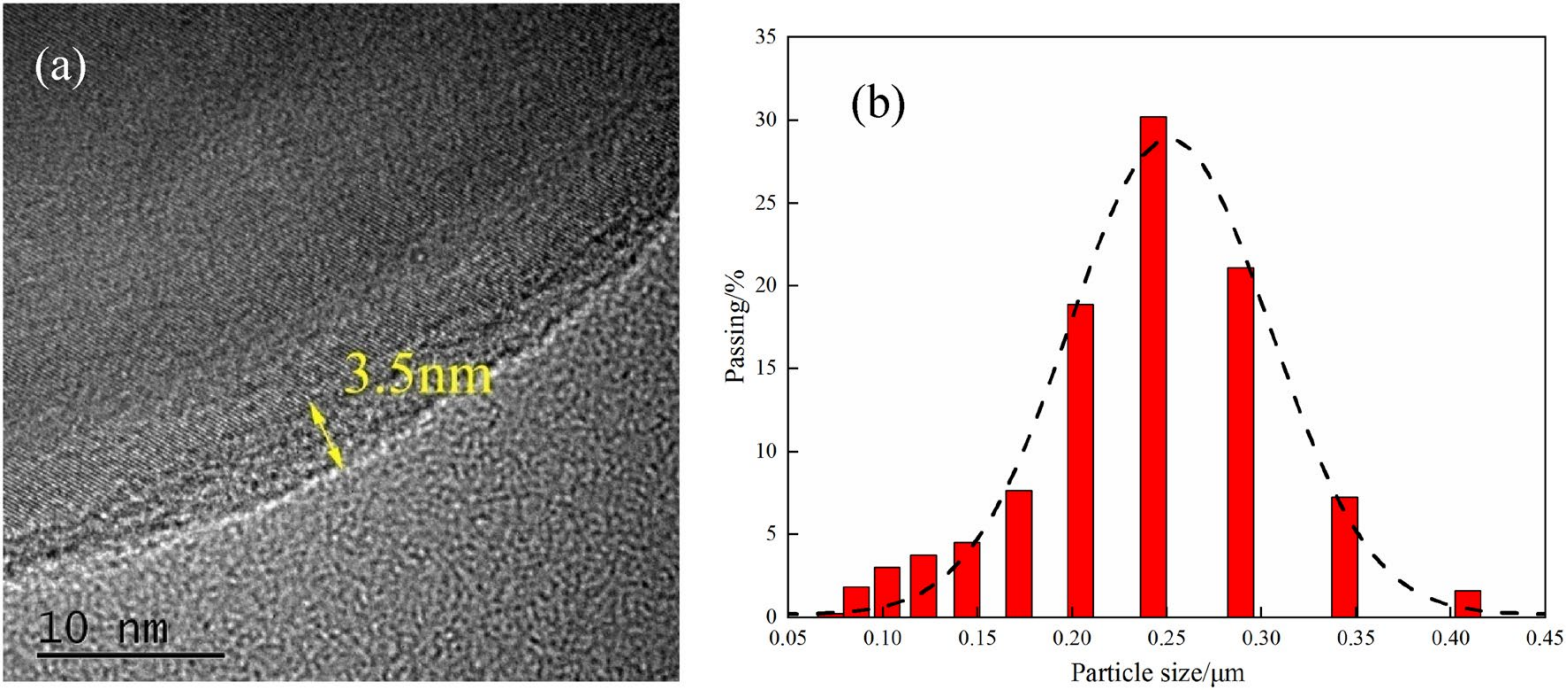
Microstructure characterization of F2311@Al: (**a**) HTEM photo; (**b**) particle size distribution.

HTEM was used to observe the core–shell structure of F2311@Al. The samples were ultrasonic dispersed to weaken the agglomeration. Then, the encapsulated samples are dispersed in ethyl alcohol and dropped on micro-grid after drying. HTEM images shown in Fig. 4a demonstrates the core–shell structure of the samples. Aluminum was clearly separated from the outer organic material. The thickness of the outer layer is 3.5 nm, which may be the amorphous oxide of Al or F2311. The thickness of the coating layer was measured by HTEM, and the result showed that the average thickness is 3.6 nm.

The particle size distribution of coating samples was characterized by dynamic light scattering (DLS) particle size analyzer. 1.0 mg samples were dispersed for 0.5 h by ultrasonic in 150 mL ethyl alcohol before the measurement. The particle size distribution shown in Fig. 4b indicates that the particle size distribution of F2311@Al basically met the normal distribution and the median particle size *D*_50_ was 217.0 nm.

### Thickness of coating layer

**Figure 5:**
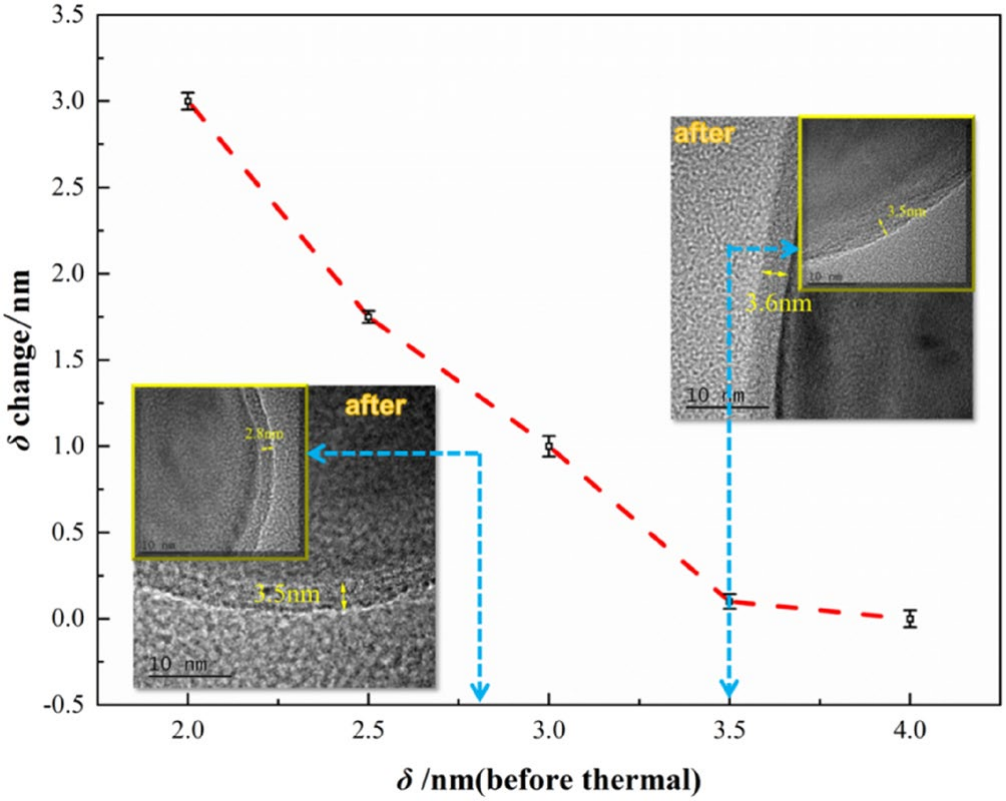
Changes in coating thickness before and after heated.

The thickness of coating could significantly affect their application. Thus, the coating should be as thin as possible to assure the high energy release. However, the storage safety of the samples would be better with a thicker coating. Aluminum powders with different coating thickness could be prepared by changing the mass ratio of F2311 and aluminum. In order to investigate the optimal coating thickness, the thermal stability of F2311@Al was tested at 75 °C for 48 h according to the national military standard GJB 5891.13–2006. First, 2 g of F2311@Al were heated at 75 °C for 48 h. Then, the thickness of coating layer was measured by TEM. The critical thickness of coating was deduced by comparing the changes of coating thickness before and after the test. The alumina thickness of raw aluminum is about 3.8–4.0 nm. As is shown in Fig. 5, when the coating thickness is about 3.6 nm, it barely increased after being heated. When the coating thickness is less than 3 nm, the shell (coating and alumina) increased significantly after being heated, indicating that the coating is not dense when less than 3 nm, which means oxygen in the air can diffuse through the shell easily to trigger the slow oxidation on core–shell interface. And the growth of oxide will result in the shell increasing after heated. Therefore, the critical thickness of F2311 is 3.6 nm.

**Figure 6:**
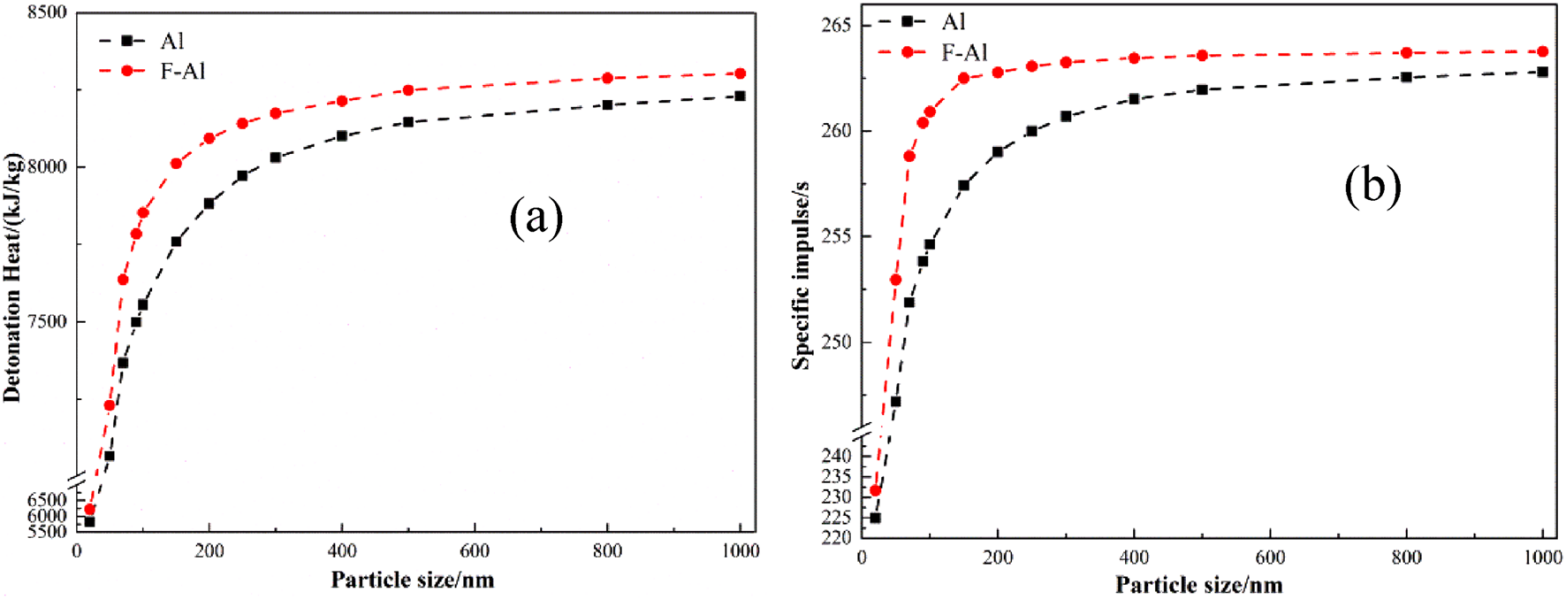
Calculation results of detonation heat and specific impulse of F2311@Al and ordinary Al: (**a**) detonation heat; (**b**) the specific impulse.

Based on the above analysis, the parameters of F2311@Al were confirmed, namely, the median particle size of aluminum powder is about 200 nm, and the thickness of F2311 is 3.60 nm. In order to figure out the amount of ordinary aluminum powder and F2311@Al that could affect energetic composites, EXPLO5 6.03 was applied to calculate the combustion and explosion parameters (theoretical specific impulse and ideal detonation heat) of aluminum-containing energetic composites under the same condition based on BKW equation^39,40^ and the principle of minimum free energy^41^. When calculating the detonation heat, we assumed that the system insists of 80 wt% CL-20 and 20  wt% Al^42^. When calculating the adiabatic constant pressure combustion, the system is composed of 15  wt% HTPB, 65  wt% AP, and 20%wt Al^43^. Al represents F2311@Al and ordinary Al, respectively. The F2311 in F2311@Al and alumina in ordinary Al share the same mass ratio. Because the density of fluororubber (1.85 g/cm^3^) is less than that of alumina (2.98 g/cm^3^), the thickness of F2311 is larger and denser. The results are shown in Fig. 6.

Figure 6a is the calculation result of detonation heat. It can be seen that within the range of 20–1000 nm, the detonation heat of F231@Al is higher than that of raw Al. The detonation heat of the system increases with the increase of particle size, which is due to the more active aluminum with the growth of particle size. Since fluorine will participate in exothermic reaction, as shown in Eqs. (1) and (2), when the mass of outer shell is settled, the detonation heat of F2311@Al is higher than that of ordinary Al. Based on the calculation results, we could see that when the particle size of aluminum powder is at the nanometer scale (≤ 100 nm), the content of active aluminum is relatively low, so the detonation heat is not low. When the size of aluminum powder is between 100 and 400 nm, the detonation heat of the two aluminum powders is significantly different, especially in the vicinity of 200 nm. When the particle size is above 400 nm, the detonation heat of the system does not increase significantly, and the detonation heat tends to be stable, which is gradually close to the calculation results of micron aluminum powder. Thus, it is confirmed that the aluminum powder with a median particle size of 200 nm used for encapsulated in-situ has excellent potential of releasing energy in the energetic composites. The calculation results of theoretical specific impulse show similar trends. When the particle size of aluminum powder is within the range of 100–400 nm, the theoretical specific impulse’s difference is the largest. The formula of F2311@Al is significantly higher than that of ordinary Al, and the specific impulse remains unchanged after 400 nm. In conclusion, when the average particle size of submicron aluminum powder is 200 nm, and the fluororubber is used for encapsulated in-situ, the combustion and detonation efficiency of the energetic composites is better, and the reactivity of products is higher accompanied with better thermal stability.

1 $$ {\text{2Al}} + {\text{3CF}}_{{2}} \to {\text{2AlF}}_{{3}} + {\text{3C}} + {\text{1481 kJ/mol}} $$

2 $$ {\text{2Al}} + {1}.{\text{5O}}_{{2}} \to {\text{Al}}_{{2}} {\text{O}}_{{3}} + {\text{455 kJ/mol}} $$

### X-ray photoelectron spectroscopy analysis

**Figure 7:**
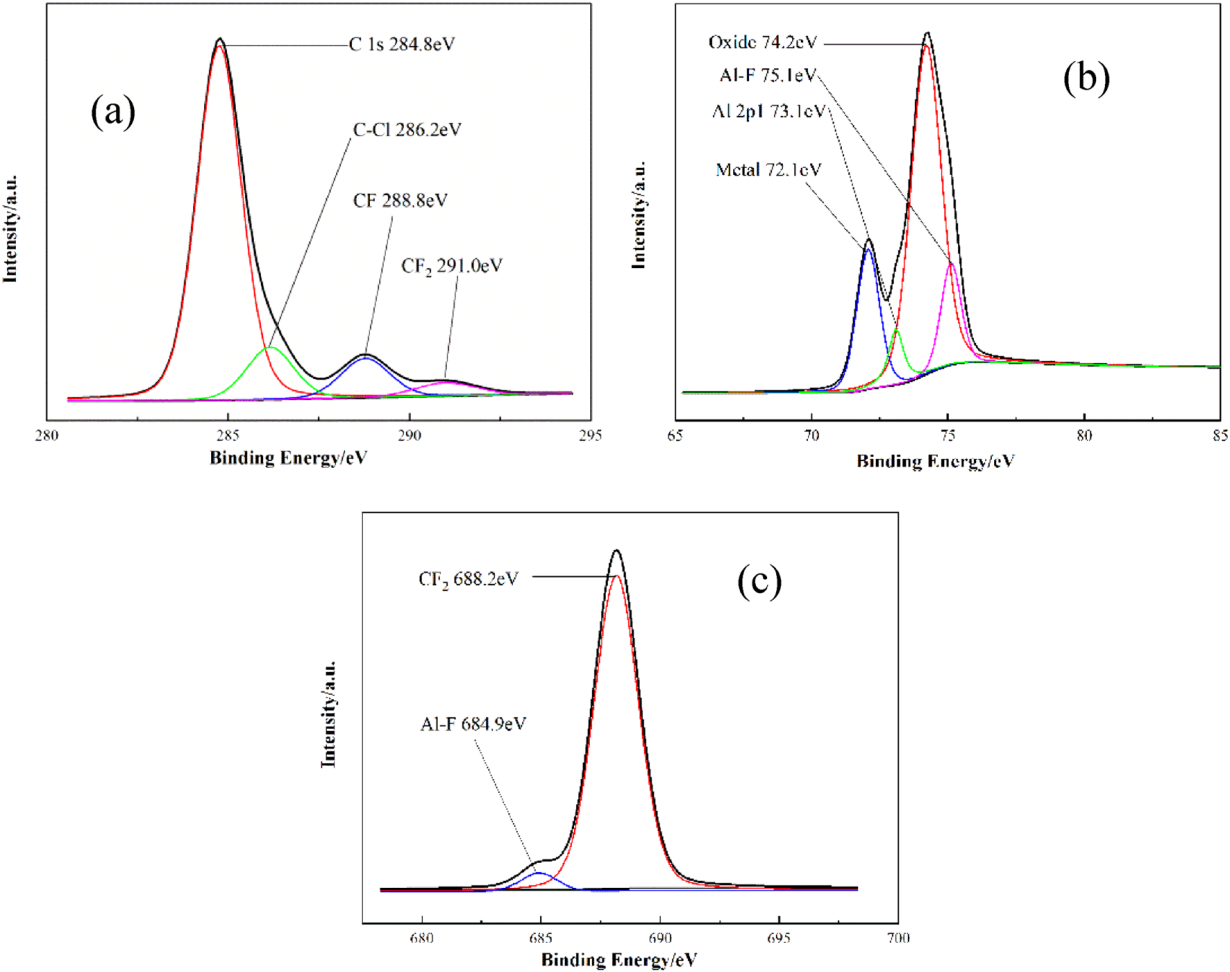
XPS spectra of F2311@Al: (**a**) 1 s orbital of C; (**b**) the 2p orbital of Al; (**c**) the 1 s orbital of F.

The results of SEM show that there is a certain amount of O on the F2311@Al coating sample. XPS were used to analyze the surface valence bond states of F2311@Al. The measured depth of XPS is 3–5 nm, which is similar to the thickness of the coating layer. Therefore, the coating layer information can be inferred. Firstly, the presence of C, O, Al and F on the surface is determined by wide-spectrum scanning. Then C, Al and F were narrow scanned, and the XPS spectra is shown in Fig. 7. Figure 7a shows that there are four chemical states in C, which are C1s (284.8 eV), C–Cl (286.2 eV), CF (288.8 eV) and CF_2_ (291.0 eV), all of which are from F2311. Figure 7b shows that there are four chemical states in Al, which are Al_2_O_3_ (74.2 eV), aluminum (72.1 eV)^44^, Al-F (75.1 eV)^45^ and Al 2p1 (73.1 eV), indicating that there are a small amount of alumina on the surface. The results of Al and F elements showed that there are Al-F bonds in the coating layer, (75.1 eV, 684.9 eV). It is speculated that there are two main ways to generate Al-F compounds. One is that Al atoms reacted with fluororubber at the interface. Another is that the alumina on the surface reacted with fluoride. Since F is more electronegative than O, F replaced O to form AlF_3_. In addition, Fig. 7c demonstrates that the peak strength of Al-F bond is much lower than CF_2_, indicating that the F2311 exists mostly as polymer, and will form chemical bonds only when it contacts with Al directly. According to the XPS results, there are F2311, AlF_3_, and a small amount of alumina on the surface of F2311@Al.

### Non-isothermal reaction kinetics

**Figure 8:**
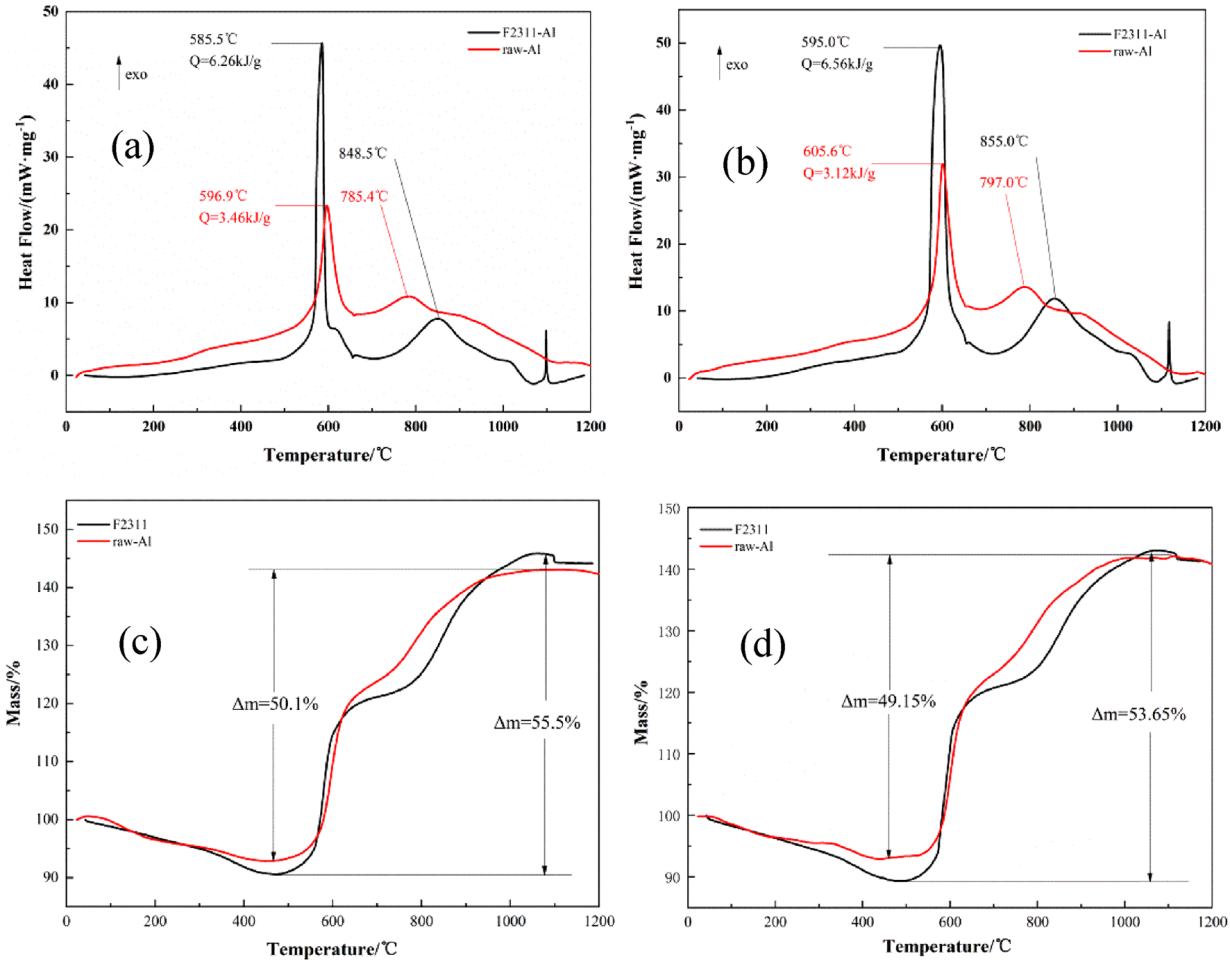
TG/DSC curves of F2311@Al and ordinary Al at different heating rates: (**a**) 10 °C/min-DSC; (**b**) 15 °C/min-DSC; (**c**) 10 °C/min-TG; (**d**) 15 °C/min–TG.

Previous studies about Al/F reaction were conducted in argon or other inert gas, while Al/F composites are usually used in open systems or in aerobic environments. Therefore, we investigated the adiabatic and non-isothermal reactions between F2311@Al and ordinary aluminum in air systematically in this study. Figure 8 shows the TG/DSC curves of two aluminum powders at different heating rates. The TG curves show that there are two stages of weight gain (550–650 °C and 750–850 °C), corresponding to the two obvious exothermic peaks in the DSC curves.

At the heating rate of 10 °C/min, the peak temperature of F2311@Al in the first stage is 11.4 °C lower than that of ordinary Al, and the heat release is 2.80 kJ/g higher. At the heating rate of 15 °C/min, the peak temperature of F2311@Al in the first stage is 10.6 °C lower, and the heat release increases by 3.44 kJ/g. The reaction temperature of F2311@Al is lower, and the heat release is around twice as high as that of ordinary aluminum. However, there is a slight delay in the reaction of F2311@Al in the second stage of weight gain. The reaction processes of two samples are quite different. After 1000 °C, the weight gain of both samples is close to 150%.

To further investigate the response mechanism of the F2311@Al and ordinary aluminum, the thermal reaction integral model functions were fitted using non-isothermal chemical reaction dynamics. Activation energy *Ea* and pre-exponential factor *A* were calculated by Ozawa method, and 41 types of kinetic model functions and *α*–*T* (*α*–*T* data is the conversion degree (*α*) varies with temperature (*T*)) data were calculated^46^. Eventually, the two stages’ most probable mechanism functions and kinetics parameters were selected and shown in Table 2. More detailed analysis could be seen in Appendix B.

**Table 2:** Kinetic parameters of exothermic reaction of F2311@Al and ordinary Al.

Sample	Stage	*Ea* (kJ/mol)	lg (A/s)^−1^	The most possible functions	Kinetic equation
F2311-Al	1st	255.10	21.96	*f(α)* = 6(1 − *α*)^2/3^[1 − (1 − *α*)^1/3^]^1/2^	d*α*/dt = 10^22.74^ × (1 − *α*)^2/3^[1 − (1 − *α*)^1/3^]^1/2^ × e^−32553.5/*T*^
	2nd	272.14	13.49	*f(α)* = (1 − *α*)^2/3^	d*α*/dt = 10^13.49^ × (1 − *α*)^2/3^ × e^−30939.4/*T*^
Raw Al	1st	299.90	24.24	*f(α)* = 1	d*α*/dt = 10^24.24^ × e^(−36071.68/*T*)^
	2nd	272.17	15.52	*f*(*α*) = 3/2 × [(1 − *α*)^−1/3^–1]^−1^	d*α*/dt = 10^15.34^ × [(1 − *α*)^−1/3^–1]^−1^ × e^(−32736.35/*T*)^

It can be seen from Table 2 that the mechanism of F2311@Al first-stage oxidation process is the Jander equation of n = 1/2, and the process is controlled by the three-dimensional diffusion rate. The integral formula is *G*(*α*) = [1 − (1 − *α*)^1/3^]^1/2^, and the corresponding differential form is *f*(*α*) = 6(1 − *α*)^2/3^[1 − (1 − *α*)^1/3^]^1/2^. At this time, the fluoride in the shell has completely decomposed, and because of the lack of a tight layer of alumina binding, aluminum particles are surrounded by "fluorine atmosphere" and hot air. The rate of Al oxidation depends on the diffusion rate of these oxidizing gases. The most probable mechanism function is differential equation *f*(*α*). The average *Ea* and *A* were substituted into the equation d*α*/dt = A*f*(*α*)e^−E/R*T*^, and the kinetic equation was obtained as d*α*/dt = 10^22.74^ × (1 − *α*)^2/3^[1 −  (1 − *α*)^1/3^]^1/2^e^−32553.5/*T*^.

The Mapel Power law is applicable to the first-stage reaction process of ordinary Al. The control step is one-dimensional phase boundary diffusion, the integral formula is *G*(*α)* = *α*, and the differential formula is *f*(*α*) = 1. This is due to the existence of alumina on the ordinary Al surface, which is relatively steady and will not participate in the reaction. Therefore, the oxidation reaction process depends on the diffusion rate of oxygen in the shell. The data in Table 2 showed that the apparent activation energy of F2311@Al in the first phase is 45 kJ/mol lower than that of ordinary Al (Ozawa method). It is reported^27^ that PVDF delayed the aluminum’s oxidation temperature. However, F2311 promoted the oxidation reaction of aluminum. Because fluorine polymer effectively reduces the content of alumina, and F is more electronegative than O. Thus, the pre-ignition reaction (PIR) will occur in the first stage, and the activation energy will decrease. Meanwhile, due to the higher reaction heat release between Al and F, the total heat release in the first stage of F2311@Al increases. In general, the encapsulated sample not only has lower activation energy, but also has higher output of heat (3.44 kJ/g higher).

The most probable mechanism function of the second-stage oxidation process of F2311@Al powder was the three-dimensional shrinkage spherical equation of n = 3, and the control step is spherically symmetric phase boundary reaction, following the deceleration type *α*-*t* curve. The integral formula is *G*(*α*) = 3[1 − (1 − *α*)^1/3^], and the corresponding differential form is *f(α)* = (1 − *α*)^2/3^. The kinetic equation is: d*α*/dt = 10^13.49^ × (1 − *α*)^2/3^e^−30939.4/*T*^. The most probable mechanism function of the second-stage oxidation process of ordinary Al is G–B equation, *G*(*α*) = 1 − 2/3 × *α* −  (1 − *α*)^2/3^, *f*(*α*) = 3/2 × [(1 − *α*)^−1/3^–1]^−1^. Reaction speed control step is spherically symmetric 3d diffusion equation, following the deceleration type *α*-*t* curve. In the second stage, the reaction activation energy and pre-exponential factor of the two aluminum powders is similar, and the kinetic mechanism function is similar, indicating that the reaction in this stage is mainly the process of oxidizing atmosphere (O and CO_2_) diffusing to Al core to form alumina.

### Al-F interface reaction mechanism

**Figure 9:**
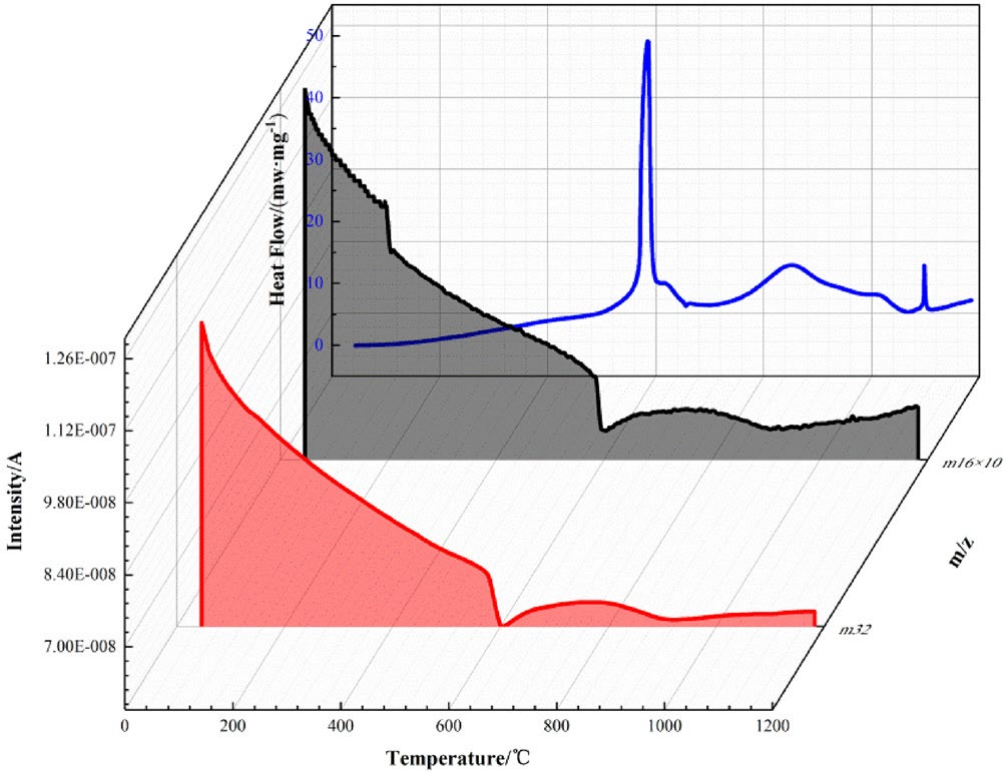
MS spectrum with O containing fragments.

**Figure10:**
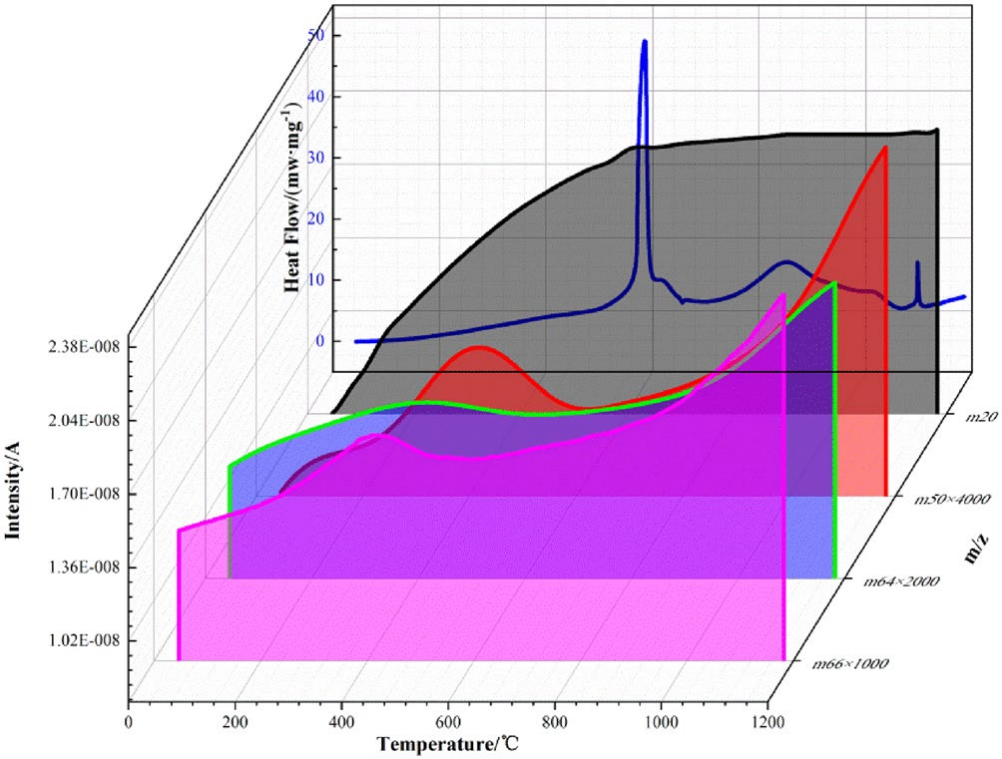
MS spectrum with F containing fragments.

In recent years, the conjecture and description of reaction mechanism of the Al-F interface have been reported. It is generally accepted as the "pre-ignition" theory, that is, before the beginning of aluminum’s oxidation, a "shucking" like solid-phase chemical reaction between alumina and fluoride will occur. In order to further reveal the details of the interface reaction of F2311@Al, the sample was tested from room temperature to 1000 °C in air at a heating rate of 10 °C/min combined with mass spectrometry (MS). Figures 9 and 10 respectively shows the mass spectrum of oxygen-containing fragments and fluorine-containing fragments.

Figure 9 shows the MS spectrum with O containing fragments. The intensity of O containing ions slowly decreases from 200 °C. This is because with the increase of temperature, oxygen is more likely adsorbed on the surface of the particles. At about 550 °C, which is the first stage of reaction, oxygen intensity falls sharply and then rebounds after 600 °C. It falls again in the second stage at about 800 °C. The two decreases in signal intensity of O containing ions correspond to the aluminum oxidation in the first and second stage.

As the MS spectrum with F containing fragments shown in Fig. 10, almost all fragments are generated between 200 and 400 °C. The signal intensity of m/z = 20 is very intense. It is speculated that this fragment is HF^+^. HF^+^ is the main product of decomposition of polyvinylidene fluoride^32^, which means that F2311 begins to decompose at 200 °C. Unlike the oxygen ions of which signal intensity decreases at first, HF^+^ remains high concentration even at 500–800 °C, when the aluminum powder began to oxidize. HF^+^ concentration does not decrease with the fluoride decomposition it is speculated that it is the product of subsequent thermal reaction.

In addition to HF fragments, there are also some fragments with large molecular weights, such as m/z of 50, 64 and 66, corresponding to CF_2_=CH_2_, F_2_C and COF_2_. Before 400 °C, these fragments occur with the decomposition of F2311 and gradually enriches, which corresponds to the slight weight loss before 400 °C on the TG curve (Fig. 8). After 400 °C, CF_2_=CH_2_, F_2_C and COF_2_ ions flow intensity decreases dramatically, while the intensity of HF ions flow is still rising. TG curve does not increase significantly in 400–550 °C. Therefore, the fragments above do not react with the Al core at this time. They just react with alumina on the surface, and alumina transforms into AlF_3_. This reaction leads to the weight loss mass in 400–550 °C. This process only occurs on the surface, and we call it pre-ignition reaction (PIR).

When system is further heated up to 580–600 °C, the reaction of aluminum initiates, and releases a large amount of heat. The signal intensity of CF_2_=CH_2_, COF_2_ and F_2_C quickly drops to the bottom. Fragments of fluoride no longer stay on the surface to participate in PIR, they go through the interface, and reacts with the Al inside. However, there is no sign of AlF_3_, which should be seen at this time in mass spectrometry. This is because AlF_3_ is still in solid phase due to its high melting point (1040 °C), and MS device only collects fragments of the gas phase. It can be seen that in the first stage (550–650 °C) of oxidation, after that they all rise up. The first weight gain reaction is more intense. We assumed that F mainly reacts with Al in the first stage. In addition, HF and other products are released due to the reaction between fluoride and aluminum, so the concentration of these fragments increased after 650 °C.

**Figure11:**
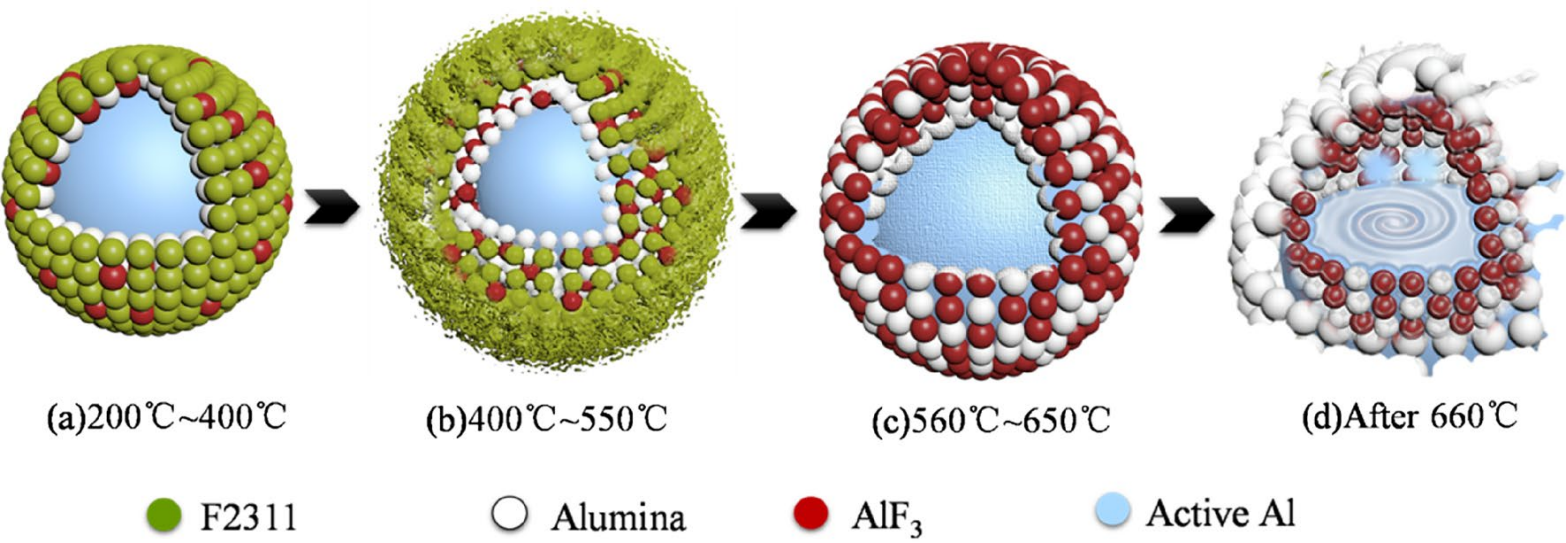
Oxidation reaction of F2311@Al.

In conclusion, according to TG-DSC-MS analysis, it is inferred that the interface reaction of F2311@Al can be divided into the following four stages:

1. Between 200 and 400 °C, oxygen slowly diffuses and is adsorbed in the coating layer, and amorphous alumina gradually thickens. At the same time, F2311 began to decompose slowly with the temperature rising, releasing small molecular monomers of vinylidene fluoride and trifluorochlor oethylene, such as CF_2_=CH_2_, COF_2_, F_2_C. The sample slightly lightens at this stage, as shown in Fig. 11a.
2. When the temperature ranging from 400 to 550 °C, the F containing fragments produced by F2311 decomposition diffuses into the alumina lattice, replacing amorphous Al_2_O_3_ with AlF_3_. Pre-ignition reaction occurs between F2311 decomposition products and Al_2_O_3_ in the coating layer to generate AlF_3_ and HF, as shown in Fig. 11b. Since the ion current strength of F-containing monomer decreases at this stage, and the weight of samples decreases, these fluorine-containing fragments do not react with the Al core, but only reacted with the alumina in the coating layer. The reaction only happens in the interface.
3. Since AlF_3_ density is less than Al_2_O_3_, the replacement of Al_2_O_3_ by AlF_3_ results in an increase of voidage on the shell, which provides a channel for oxygen diffusion. Oxygen go through the shell and diffuses to the active aluminum, triggering the first stage of oxidation. Severe oxidation of aluminum powder occurred at 560–650 °C, as shown in Fig. 11c. The first step of exothermic reaction of ordinary Al is one-dimensional phase boundary reaction, and oxygen diffusing is slow. However, the reaction speed control step of F2311@Al is three-dimensional diffusion, the activation energy is lower, the reaction rate is accelerated, so oxygen can contact the active aluminum. At the same time, some fluorine-containing fragments also contact with Al core, generating a large amount of AlF_3_. The heat release of Al-F reaction is greater than that of Al-O, so the heat release of F2311@Al in first stage is much higher than ordinary Al.
4. As the reaction proceeding, a new alumina shell regrows and again hinders oxygen diffusion and also passively absorbs heat from the system, thus ending the reaction. Aluminum melts at about 660 °C, and a very small endothermic peak can be observed on the DSC curve. Aluminum changes from solid phase to liquid phase resulting in the growth of volume, which can enlarge the shell^47^, as shown in Fig. 11d. After 800 °C, the liquid Al breaks the shell and erupts, and they quickly react with oxygen. Thus, the second exothermic peak occurs. The *Ea* and *A* of the second exothermic stage is very similar to ordinary Al, and the there’s no sign of F reacting with Al in second stage according to MS, we assumed that second exothermic reaction is mainly the reaction of Al and O_2_.

## Conclusion

In this work, we systematically studied the microstructure, surface bonding structure, thermal stability and the interface reaction mechanism of F2311 in-situ encapsulated submicron aluminum powder. The following conclusions were obtained:

1. By studying the correlation between aluminum particle size and activity, it is concluded that submicron aluminum powder (median particle size 200 nm) has the advantages of rapid reaction kinetics and high active metal content, and is a promising raw material for encapsulated in situ process. F2311 with better comprehensive performance is selected as the coating material.
2. The thermal reaction integral model functions were fitted using non-isothermal chemical reaction dynamics. The improved electrical exploding wire technology is used to prepare the F2311 in-situ encapsulated aluminum powder. We found that the appropriate coating thickness is 3.6 nm via using the thermal stability test. And F2311@Al performs better than ordinary Al in energetic composites according to the combustion and detonation calculation.
3. The thermal reaction integral model functions were fitted by using non-isothermal chemical reaction dynamics to analyze the response mechanism of the F2311@Al and ordinary aluminum. The mechanism of F2311@Al first-stage oxidation process is the Jander equation of n = 1/2 while the Mapel Power law is applicable to the first-stage reaction process of ordinary Al. The apparent activation energy of F2311@Al is 45 kJ/mol lower and can release more heat (3.44 kJ/g higher) in the first stage of oxidation. The interfacial reaction process of F2311@Al was analyzed by TG-DSC-MS. The results showed that F2311@Al not only solves the problem of deactivation and instability of nano-aluminum powder, but also accelerates the oxidation process by pre-ignition reaction between F2311 pyrolysis products and alumina. Therefore, F2311 in-situ encapusulated submicron aluminum could be promising candidate for application in energetic composites and energetic devices.

## Supplementary Information

Supplementary Information
