# Supplementary material for: A latent highly activity energetic fuel: thermal stability and interfacial reaction kinetics of selected fluoropolymer encapsulated sub-micron sized Al particles
Source: Sci Rep. 2021 Jan 12;11:738. doi: 10.1038/s41598-020-80865-2 (PMC7804492; doi:10.1038/s41598-020-80865-2)
Supplement: Supplementary file 1 — Supplementary Information [file 41598_2020_80865_MOESM1_ESM.docx]

A latent highly activity energetic fuel: thermal stability and interfacial reaction kinetics of selected fluoropolymer encapsulated sub-micron sized Al particles

Huixin Wang^1^, Hui Ren^1,^*, Tao Yan^1^, Yaru Li^1^ ,and Wanjun Zhao^1^

^1^State Key Laboratory of Explosion Science and Technology, Beijing Institute of Technology, Beijing, 100081, China

^*^renhui@bit.edu.cn

**Appendix A**

In order to determine the optimal particle size of aluminum powder, the thickness of oxide layer of naturally oxidized aluminum powder with different particle sizes was measured. During the measurement, the TEM photos of aluminum powder were processed by Digital Micrograph. The complete and clear outer and inner diameters of aluminum powder in the TEM photos were measured once per 72° rotation. The thickness of the oxide layer was obtained by subtracting the outer diameter from the inner diameter. The average value was taken for 5 times. The results are listed in Table A.1.

**Table A.1** Thickness of oxide layer of raw aluminum powder

| ^Particle size/nm^ | ^Thickness/ standard deviation^ | ^Active aluminum content (%)^ |  | ^Particle size/nm^ | ^Thickness/ standard deviation^ | ^Active aluminum content (%)^ |
| --- | --- | --- | --- | --- | --- | --- |
| ^25^ | ^2.995/0.0187^ | ^41.5^ |  | ^100^ | ^3.494/0.0416^ | ^78.81^ |
| ^30^ | ^3.043/0.0269^ | ^48.13^ |  | ^120^ | ^3.643/0.0490^ | ^81.36^ |
| ^35^ | ^3.020/0.0198^ | ^54.15^ |  | ^150^ | ^3.703/0.0369^ | ^84.61^ |
| ^40^ | ^3.011/0.0165^ | ^58.87^ |  | ^200^ | ^3.838/0.0435^ | ^87.86^ |
| ^45^ | ^3.003/0.0179^ | ^62.73^ |  | ^250^ | ^3.924/0.0262^ | ^89.97^ |
| ^50^ | ^3.007/0.0146^ | ^65.84^ |  | ^300^ | ^4.044/0.0203^ | ^91.33^ |
| ^55^ | ^3.004/0.0162^ | ^68.54^ |  | ^350^ | ^4.067/0.0148^ | ^92.48^ |
| ^60^ | ^2.972/0.0291^ | ^71.09^ |  | ^400^ | ^4.092/0.0271^ | ^93.35^ |
| ^65^ | ^3.056/0.0483^ | ^72.38^ |  | ^450^ | ^4.183/0.0140^ | ^93.94^ |
| ^70^ | ^2.990/0.0349^ | ^74.62^ |  | ^500^ | ^4.227/0.0450^ | ^94.47^ |
| ^75^ | ^3.196/0.0238^ | ^74.68^ |  | ^550^ | ^4.221/0.0385^ | ^94.96^ |
| ^87.5^ | ^3.384/0.0592^ | ^76.78^ |  | ^600^ | ^4.275/0.0354^ | ^95.31^ |

Equation (A.1) is the correlation of shell thickness and particles$\delta=\left\{\begin{matrix} 3.022\text{ }d\leq70 \\ 3.621\cdot\left(1-\text{e}^{-0.0248d}\right)+1.251\cdot\left(1-\text{e}^{-0.0012d}\right)\text{ 70}\leq d\leq600 \end{matrix}\right.$ (A.1)

**Appendix B**

Five analytical methods(Flynn-Wall-Ozawa method^4849^,Ordinary-Integral method^50^, Šatava-Šesták method^51^, Coat –Redfern method^50^ and Agrawal method^52^) were used to calculate F2311 coating aluminum powder reaction kinetics parameters at different heating rates rate (10, 15, 20 and 25 K·min^-1^).







(a) (b)

**Figure B.1** TG(a) and DSC(b) curves of F2311 coating aluminum at different heating rates







(a) (b)

**Figure B.2.** TG curves of F2311 coating aluminum powder at different stages :(a) the first stage and (b) the second stage

Figure B.2. shows TG curves of the first and second stages. Each of the TG curves is approximately a straight line under the heating rate and is divided into 10 parts on average.1, read the corresponding temperature *T.* The *α*-*T* data obtained from Figure 11 are substituted into the mechanism function, different *Ea*, lgA, r and Q were obtained. Where, *Ea* is apparent active energy, *A* is pre-exponential factor, r is the correlation coefficient and Q is the deviation. According to the optimal principle of r and Q, the corresponding kinetic parameters of 41 mechanism functions were calculated. Then according to the principle that Ea and lgA are basically consistent with the result of Ozawa method, the results were obtained.

In order to compare the effect of F2311 coating on the reaction of aluminum powder, non-isothermal reaction kinetics was carried out for raw aluminum powder. The heating rates adopted were 5, 7.5, 10 and 15K·min^-1^. Figure IV shows TG and DSC curves of raw aluminum powder under different heating rates. The mechanism functions of the first two stages of raw aluminum powder are deduced







(a) (b)

**Figure B.3.** TG(a) and DSC(b) curves of raw aluminum at different heating rates

Table B.1 and Table B.2 are the kinetic parameters of the thermal reaction of F2311 coating submicron aluminum powder and ordinary aluminum powder.,

**Table B.1** Kinetic parameters of the first and second stages of F2311 coated samples

| ^Methods^ | ^β/(K·min-1)^ | ^The first stage^ | | | | ^The second stage^ | | | |  |
| --- | --- | --- | --- | --- | --- | --- | --- | --- | --- | --- |
|  |  | ^Ea(kJ/mol)^ | ^lg(A/s-1)^ | ^r^ | ^Q^ | ^Ea(kJ/mol)^ | ^lg(A/s-1)^ | ^r^ | ^Q^ | |
| ^Ordinary-Integral^ | ^10^ | ^289.46^ | ^24.05^ | ^0.9863^ | ^0.0599^ | ^277.10^ | ^14.86^ | ^0.9870^ | ^0.2413^ | |
|  | ^15^ | ^272.78^ | ^22.237^ | ^0.9835^ | ^0.0713^ | ^248.42^ | ^12.89^ | ^0.9865^ | ^0.2466^ | |
|  | ^20^ | ^252.21^ | ^20.26^ | ^0.9823^ | ^0.0762^ | ^243.25^ | ^12.75^ | ^0.9859^ | ^0.2586^ | |
|  | ^25^ | ^272.08^ | ^21.63^ | ^0.9832^ | ^0.0724^ | ^259.25^ | ^13.45^ | ^0.9872^ | ^0.2346^ | |
| ^Šatava-Šesták^ | ^10^ | ^284.41^ | ^23.66^ | ^0.9871^ | ^0.0108^ | ^276.77^ | ^14.82^ | ^0.9882^ | ^0.0449^ | |
|  | ^15^ | ^268.71^ | ^21.91^ | ^0.9846^ | ^0.0129^ | ^249.79^ | ^12.95^ | ^0.9880^ | ^0.0457^ | |
|  | ^20^ | ^249.26^ | ^20.00^ | ^0.9836^ | ^0.0137^ | ^244.78^ | ^12.82^ | ^0.9873^ | ^0.0480^ | |
|  | ^25^ | ^268.34^ | ^21.33^ | ^0.9844^ | ^0.0131^ | ^260.35^ | ^13.49^ | ^0.9885^ | ^0.0435^ | |
| ^Coats-Redfern^ | ^10^ | ^289.46^ | ^24.03^ | ^0.9862^ | ^0.0572^ | ^277.10^ | ^14.83^ | ^0.9870^ | ^0.2382^ | |
|  | ^15^ | ^272.78^ | ^22.21^ | ^0.9835^ | ^0.0682^ | ^248.42^ | ^12.85^ | ^0.9865^ | ^0.2428^ | |
|  | ^20^ | ^252.22^ | ^20.23^ | ^0.9823^ | ^0.0726^ | ^243.25^ | ^12.71^ | ^0.9859^ | ^0.2545^ | |
|  | ^25^ | ^272.08^ | ^21.60^ | ^0.9832^ | ^0.0693^ | ^259.25^ | ^13.41^ | ^0.9872^ | ^0.2310^ | |
| ^Agrawal^ | ^10^ | ^289.46^ | ^24.05^ | ^0.9862^ | ^0.0594^ | ^277.10^ | ^14.86^ | ^0.9870^ | ^0.2405^ | |
|  | ^15^ | ^272.78^ | ^22.24^ | ^0.9835^ | ^0.0707^ | ^248.42^ | ^12.88^ | ^0.9865^ | ^0.2454^ | |
|  | ^20^ | ^252.22^ | ^20.26^ | ^0.9823^ | ^0.0754^ | ^243.25^ | ^12.74^ | ^0.9859^ | ^0.2574^ | |
|  | ^25^ | ^272.08^ | ^21.62^ | ^0.9832^ | ^0.0718^ | ^259.25^ | ^13.45^ | ^0.9872^ | ^0.2336^ | |
| ^Average^ |  | ^270.65^ | ^21.96^ |  |  | ^257.23^ | ^13.49^ |  |  | |
| ^Flynn-Wall-Ozawa^ |  | ^255.10^ |  | ^0.9981^ |  | ^272.14^ |  | ^0.9492^ |  | |

**Table B.2** Kinetic parameters of the first and second stages of ordinary nano-aluminum powder

| ^Methods^ | ^β/(K·min-1)^ | ^The first stage^ | | | | ^The second stage^ | | | |  |
| --- | --- | --- | --- | --- | --- | --- | --- | --- | --- | --- |
|  |  | ^Ea(kJ/mol)^ | ^lg(A/s-1)^ | ^r^ | ^Q^ | ^Ea(kJ/mol)^ | ^lg(A/s-1)^ | ^r^ | ^Q^ | |
| ^Ordinary-Integral^ | ^5^ | ^298.40^ | ^24.23^ | ^0.9565^ | ^0.3893^ | ^259.36^ | ^14.59^ | ^0.9949^ | ^0.2577^ | |
|  | ^7.5^ | ^291.38^ | ^23.30^ | ^0.9604^ | ^0.3561^ | ^286.84^ | ^15.95^ | ^0.9931^ | ^0.3487^ | |
|  | ^10^ | ^320.26^ | ^25.72^ | ^0.9545^ | ^0.4101^ | ^291.57^ | ^16.00^ | ^0.9866^ | ^0.6744^ | |
|  | ^15^ | ^306.22^ | ^24.15^ | ^0.9503^ | ^0.4467^ | ^289.46^ | ^15.69^ | ^0.9823^ | ^0.8828^ | |
| ^Šatava-Šesták^ | ^5^ | ^293.03^ | ^23.82^ | ^0.9592^ | ^0.0728^ | ^258.23^ | ^14.49^ | ^0.9954^ | ^0.0475^ | |
|  | ^7.5^ | ^286.51^ | ^22.94^ | ^0.9628^ | ^0.0666^ | ^284.85^ | ^15.80^ | ^0.9937^ | ^0.0646^ | |
|  | ^10^ | ^314.07^ | ^25.28^ | ^0.9569^ | ^0.0769^ | ^289.61^ | ^15.86^ | ^0.9878^ | ^0.1257^ | |
|  | ^15^ | ^300.90^ | ^23.77^ | ^0.9529^ | ^0.0839^ | ^287.84^ | ^15.56^ | ^0.9839^ | ^0.1648^ | |
| ^Coats-Redfern^ | ^5^ | ^298.40^ | ^24.21^ | ^0.9565^ | ^0.3865^ | ^259.36^ | ^14.56^ | ^0.9949^ | ^0.2544^ | |
|  | ^7.5^ | ^291.38^ | ^23.28^ | ^0.9604^ | ^0.3535^ | ^286.84^ | ^15.92^ | ^0.9931^ | ^0.3461^ | |
|  | ^10^ | ^320.26^ | ^25.70^ | ^0.9545^ | ^0.4082^ | ^291.57^ | ^15.98^ | ^0.9866^ | ^0.6719^ | |
|  | ^15^ | ^306.22^ | ^24.13^ | ^0.9503^ | ^0.4450^ | ^289.46^ | ^15.66^ | ^0.9823^ | ^0.8803^ | |
| ^Agrawal^ | ^5^ | ^298.40^ | ^24.23^ | ^0.9565^ | ^0.3888^ | ^259.36^ | ^14.59^ | ^0.9949^ | ^0.2569^ | |
|  | ^7.5^ | ^291.38^ | ^23.30^ | ^0.9604^ | ^0.3556^ | ^286.84^ | ^15.94^ | ^0.9931^ | ^0.3481^ | |
|  | ^10^ | ^320.26^ | ^25.72^ | ^0.9545^ | ^0.4098^ | ^291.57^ | ^16.00^ | ^0.9866^ | ^0.6738^ | |
|  | ^15^ | ^306.22^ | ^24.15^ | ^0.9503^ | ^0.4465^ | ^289.46^ | ^15.68^ | ^0.9823^ | ^0.8821^ | |
| ^Average^ |  | ^302.71^ | ^24.24^ |  |  | ^281.39^ | ^15.52^ |  |  | |
| ^Flynn-Wall-Ozawa^ |  | ^299.90^ |  | ^0.9952^ |  | ^272.17^ |  | ^0.9969^ |  | |
